# Supplementary material for: Venomix: a simple bioinformatic pipeline for identifying and characterizing toxin gene candidates from transcriptomic data
Source: PeerJ. 2018 Jul 31;6:e5361. doi: 10.7717/peerj.5361 (PMC6074769; doi:10.7717/peerj.5361)
Supplement: Supplemental Information 6 [file peerj-06-5361-s006.gz › FinalOutput_1E-20/Venom_allergen_5_15/finaltree.pdf]

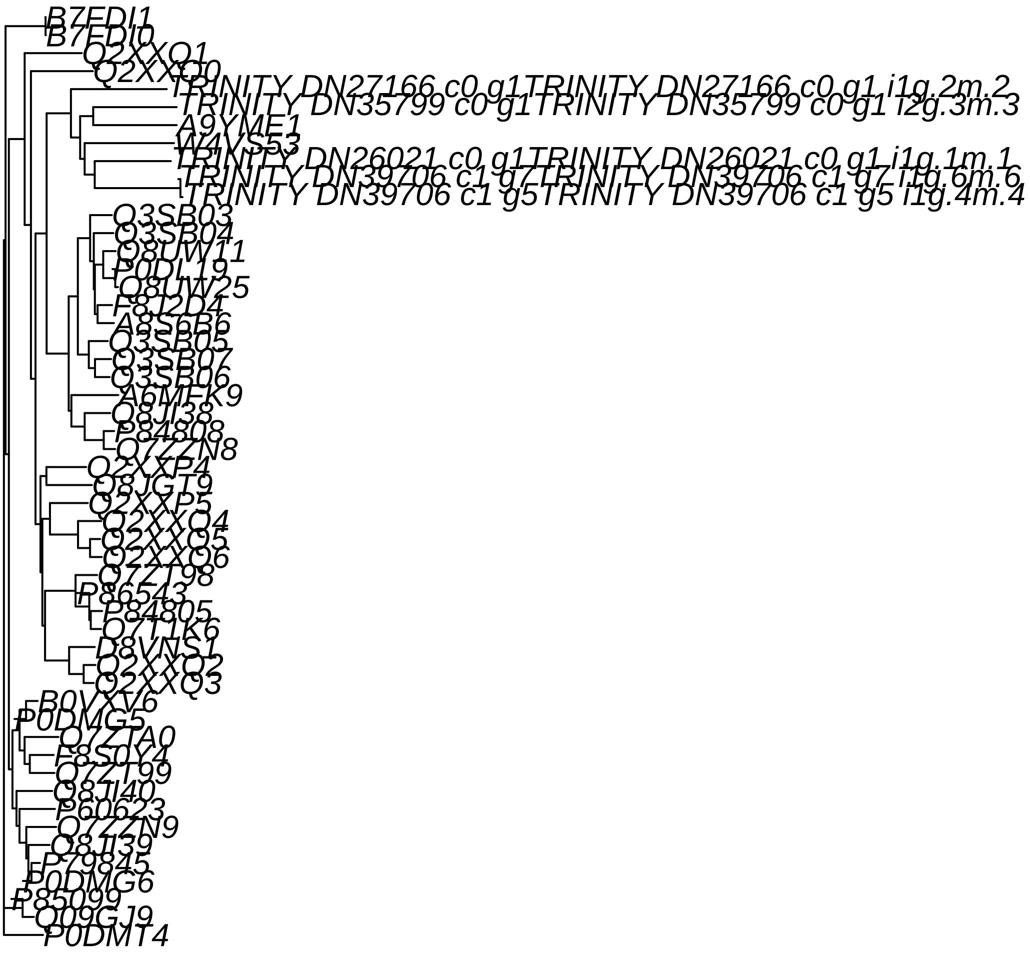

B7FDI1
B7FDI0
Q2XXQ1
Q2XXQ0
TRINITY DN27166 c0 g1TRINITY DN27166 c0 g1 i1g.2m.2
TRINITY DN35799 c0 g1TRINITY DN35799 c0 g1 i2g.3m.3
A9YME1
W4VS53
TRINITY DN26021 c0 g1TRINITY DN26021 c0 g1 i1g.1m.1
TRINITY DN39706 c1 g7TRINITY DN39706 c1 g7 i1g.6m.6
TRINITY DN39706 c1 g5TRINITY DN39706 c1 g5 i1g.4m.4
Q3SB03
Q3SB04
Q8UW11
P0DL19
Q8UW25
F8J2D4
A8S6B6
Q3SB05
Q3SB07
Q3SB06
A6MFK9
Q8JI38
P84808
Q7ZZN8
Q2XXP4
Q8JGT9
Q2XXP5
Q2XXQ4
Q2XXQ5
Q2XXQ6
Q7ZT98
P86543
P84805
Q7T1K6
D8VNS1
Q2XXQ2
Q2XXQ3
B0VXV6
P0DMG5
Q7ZTA0
F8S0Y4
Q7ZT99
Q8JI40
P60623
Q7ZZN9
Q8JI39
P79845
P0DMG6
P85099
Q09GJ9
P0DMT4
